# Targeting Metabolic Pathways of Myeloid Cells Improves Cancer Immunotherapy

**DOI:** 10.3389/fcell.2021.747863

**Published:** 2021-12-20

**Authors:** Jianying Li, Chelsea Bolyard, Gang Xin, Zihai Li

**Affiliations:** ^1^ Pelotonia Institute of Immuno-Oncology, the Ohio State University Comprehensive Cancer Center—Arthur G. James Cancer Hospital and Richard J. Solove Research Institute, Columbus, OH, United States; ^2^ Department of Microbial Infection and Immunity, the Ohio State University College of Medicine, Columbus, OH, United States; ^3^ Department of Medical Oncology, the Ohio State University College of Medicine, Columbus, OH, United States

**Keywords:** myeloid cells, immunometabolism, immunotherapy, tumor-associated macrophages, tumor-infiltrating myeloid cells, tumor-associated dendritic cells, myeloid-derived suppressor cells, tumor-associated neutrophils

## Abstract

Tumor-infiltrating myeloid cells are a prominent pro-tumorigenic immune cell population that limit host anti-tumor immunity and present a significant obstacle for many cancer immunotherapies. Targeting the mechanisms regulating myeloid cell function within the tumor microenvironment may overcome immunotherapy resistance in some cancers. Recent discoveries in the emerging field of immunometabolism reveal that the metabolic profiles of intratumoral myeloid cells are rewired to adapt to the nutrition-limited tumor microenvironment, and this shapes their pro-tumor phenotypes. Interestingly, metabolic modulation can shift these myeloid cells toward the immune-stimulating anti-tumor phenotype. In this review, we will highlight the roles of specific metabolic pathways in the activation and function of myeloid cells, and discuss the therapeutic value of metabolically reprogramming myeloid cells to augment and improve outcomes with cancer immunotherapy.

## Introduction

The tumor microenvironment (TME) is often infiltrated by a large number of myeloid cells, which represent a prominent immune component in tumors and play a critical role in modulating anti-tumor immunity (De Vlaeminck et al., 2016). The tumor-infiltrating myeloid cells (TIMs) are a heterogeneous population, including tumor-associated macrophages (TAMs), tumor-associated dendritic cells (TADCs), tumor-associated neutrophils (TANs), and myeloid-derived suppressor cells (MDSCs) (Laoui et al., 2014; Maenhout et al., 2014). Many of these TIMs originate from hematopoietic progenitors, which are recruited to the TME in response to tumor-derived chemokines and cytokines. In addition to cancer-induced myelopoiesis, TAMs can also derive from tissue-resident macrophages of embryonic origin. TIMs such as M1-like TAMs, N1-like TANs, and CD103 + TADCs can behave in an anti-tumorigenic manner and produce pro-inflammatory molecules, including tumor necrosis factor-α (TNFα) and B7-2 (CD86). However, in established tumors the majority of TIMs acquire a pro-tumorigenic phenotype that suppresses anti-tumor response and promotes tumor growth, such as MDSCs, M2-like TAMs, TANs, and TADCs. They produce an array of molecules such as arginase 1 (ARG1) and indoleamine 2,3-dioxygenase (IDO) that promote immunosuppression, and growth factors such as transforming growth factor-β (TGF-β) and vascular endothelial growth factor (VEGF) that promote metastasis and angiogenesis, respectively. The presence and accumulation of pro-tumorigenic TIMs correlates with resistance to various cancer treatments (including immunotherapy) and poor patient outcomes (Sharma et al., 2017). Thus, reprogramming TIMs towards an anti-tumorigenic phenotype is an attractive strategy for cancer therapy that has shown efficacy across therapeutic modalities and tumor types in both preclinical and clinical studies (Kowal et al., 2019; Molgora and Colonna, 2021).

**FIGURE 1:**
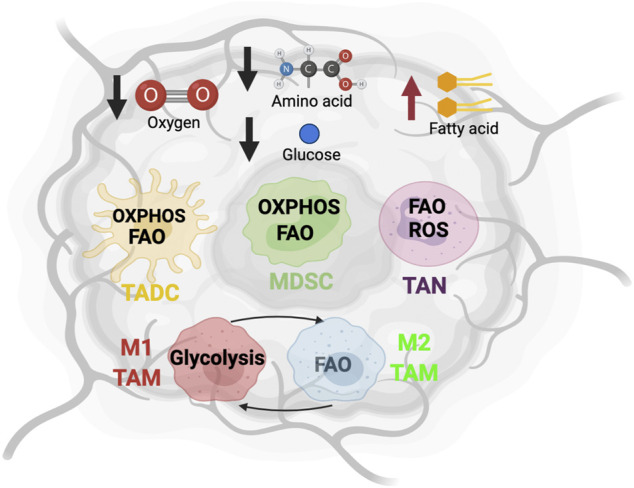
The metabolic state and immune cells in tumor microenvironment. The altered metabolic activity of tumor cells creates an environment lacking oxygen, glucose and amino acid, and enriched with fatty acids. To adapt to this nutrient challenge, myeloid cells such as tumor-associated dendritic cells (TADC), myeloid-derived suppressor cells (MDSC), tumor-associated neutrophils (TAN), and tumor-associated macrophage (TAM) alter their metabolic program.

The pro- and anti-tumorigenic phenotypes of TIMs are highly regulated by various mechanisms such as tumor-derived cytokines, growth factors, and metabolic composition. Among these environmental cues, emerging studies suggest that metabolites are one of the decisive factors driving the differentiation and function of TIMs (Hu et al., 2020a; Sieow et al., 2018). A hallmark of malignancy is metabolic reprogramming to support proliferation. In general, cancer cells exhibit the “Warburg effect” by preferentially consuming glucose and producing lactate byproduct even in presence of oxygen. Additionally, elevated rates of glutaminolysis by cancer cells facilitates the tricarboxylic acid (TCA) cycle, whose byproducts are redirected into biosynthetic reactions. A recent study used radiotracer 18F-fluorodeoxyglucose and PET imaging to characterize the nutrient uptake profile of tumor-infiltrating cells (Reinfeld et al., 2021). They reported that cancer cells consume approximately 60% of all intratumoral glucose and TIMs consume approximately 30%, while other immune cells have negligible metabolic impact (Reinfeld et al., 2021). By contrast, uptake of glutamine is dominated by cancer cells (Reinfeld et al., 2021). Furthermore, the TME is enriched with free fatty acids (FFAs) that mediate membrane biogenesis, energy production, protein modification, and signaling molecules. Cancer cells contribute to FFA abundance *via de novo* fatty acids synthesis, which leads to the accumulation of cancer-associated fibroblasts and adipocytes (Koundouros and Poulogiannis, 2020). Meanwhile, the dysfunctional tumor-associated vasculature insufficiently oxygenates the TME, which leads to the formation of hypoxic regions. Collectively, cancer cells induce a metabolically-challenging environment with limited glucose, glutamine, and oxygen, and excessive lactate and lipids (Figure 1) (Griguer et al., 2005).

Intratumoral conditions are dramatically different from the native environment of myeloid cells. Therefore, they have to undergo robust metabolic reprogramming to adapt and survive. Emerging evidence indicates that this process significantly changes the differentiation and function of TIMs. Metabolic targeting of TIMs has shown anti-tumor efficacy across several therapeutic modalities in treating various types of tumor. Here, we provide a summary of current literature on the metabolic regulation of TIMs, including how reprogramming immunosuppressive TIMs may promote anti-tumor activity. We will briefly describe the intratumoral metabolic profile and its impact on each TIM subset. Finally, we provide an overview of clinical trials designed to study the impact of metabolic modulation of TIMs, either as a novel immunotherapy or in combination with current cancer therapies.

## MDSC

**FIGURE 2:**
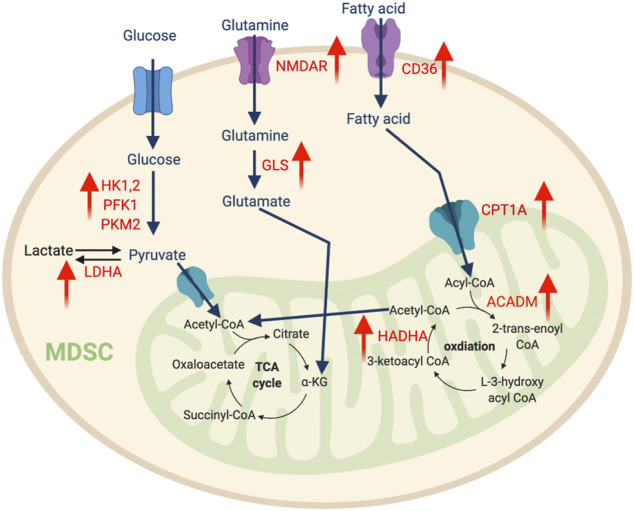
The metabolic features of MDSCs. The tumor-infiltrating MDSCs upregulate glycolysis pathway to support their expansion, while promoting amino acid and fatty acid metabolism to support their immunosuppressive function. “↑” indicates “increase.” HK, hexokinase; PFK1, Phosphofructokinase 1; PKM2, Pyruvate kinase isozymes M1/M2; NMDAR, N-methyl-d-aspartate receptor; LDHA, Lactate dehydrogenase A; GLS, Glutaminase; CPT1A, Carnitine Palmitoyltransferase 1A; HADHA, Hydroxyacyl-CoA Dehydrogenase Trifunctional Multienzyme Complex Subunit Alpha; ACADM, Acyl-CoA Dehydrogenase Medium Chain.

MDSCs originate from bone marrow-derived immature myeloid cells, which are also the precursors to dendritic cells (DCs), macrophages, and neutrophils (Tcyganov et al., 2018). During cancer-induced myelopoiesis, these precursor cells are recruited to the TME and maintain their immature differentiation state. Under the influence of tumor-derived cytokine and growth factors [e.g., VEGF, granulocyte macrophage colony stimulating factor (GM-CSF), macrophage colony-stimulating factor (M-CSF), TGF-β, IL-4, IL-6, and IL-10], MDSCs acquire inhibitory activity against various anti-tumor immune cells. MDSCs include a wide range of granulocytic and monocytic cell types at different stages of differentiation. Monocytic MDSCs (M-MDSCs) share certain phenotypic and morphological characteristics with monocytes, and can further differentiate into TAMs (Bronte et al., 2016), whereas Polymorphonuclear (PMN)-MDSCs are similar to immature neutrophils and TANs (Bronte et al., 2016). Due to their immunosuppressive features, the accumulation of MDSCs correlates with accelerated tumor progression, immune escape, and resistance to immunotherapy, which leads to poor clinical outcomes (Li et al., 2021). Therefore, metabolic regulation of MDSC function remains an area of active research (Figure 2).

### Glucose Metabolism

The enhanced aerobic glycolysis seen in tumors impacts the development of MDSCs. In this metabolic process, cells uptake glucose from extracellular environments and convert it into two molecules of ATP by a series of enzymatic reactions. Recent transcriptomic analysis of a triple-negative breast cancer dataset (Curtis et al., 2012) illustrated that elevated gene expression of multiple key glycolytic enzymes, including lactate dehydrogenase A (LDHA), hexokinase-1 (HK1), and pyruvate kinase muscle isozyme 2 (PKM2) positively correlated with increased abundance of intratumoral MDSC (Li et al., 2018). Interestingly, tumor glycolysis activates lactate dehydrogenase (LDH) *via* CCAAT/enhancer-binding protein beta and induces the production of GM-CSF and granulocyte CSF (G-CSF), which promotes emergency myelopoiesis in bone marrow (Li et al., 2018). Furthermore, tumor glycolysis also produces excessive lactate that fosters the differentiation and expansion of immunosuppressive MDSC (Husain et al., 2013; Brand et al., 2016). Dynamic metabolic flux analysis of BM-derived MDSCs showed that an increase of both glycolysis and glutaminolysis are associated with MDSC differentiation, thus indicating a Warburg effect akin to cancer cells (Goffaux et al., 2017). In addition, similar metabolic flexibility has been demonstrated in PMN-MDSCs from a nasopharyngeal carcinoma tumor-bearing mouse model (Cai et al., 2017). Indeed, another study confirmed that several glycolytic genes and glycolysis rates are augmented by the TME to support the expansion of MDSCs. This is largely attributed to the glycolytic metabolite phosphoenolpyruvate (PEP), which serves as a critical antioxidant to protect MDSCs from reactive oxygen species (ROS)-mediated apoptosis (Jian et al., 2017). Overall, glycolysis supports MDSC development and accumulation in the TME.

Although we still lack a detailed understanding of mechanisms directing the MDSC metabolic profile, several pathways have been shown to play important roles. For example, the mammalian target of rapamycin (mTOR) pathway induces the expression of glycolysis-related enzymes including HK1, hexokinase-2 (HK2), phosphofructokinase-1 (PFK1), pyruvate kinase M2 (PKM2), and lactate dehydrogenase (LDH), which are vital for M-MDSC lineage commitment (Uehara et al., 2019). Inhibition or genetic ablation of mTOR impedes glucose utilization and hinders differentiation of MDSCs from hematopoietic progenitors. Metformin, a pharmacologic glycolysis activator, can rescue this phenotype (Uehara et al., 2019). Additionally, intratumoral hypoxia activates hypoxia-inducible factor-1α (HIF-1α), which can transcriptionally induce genes associated with glucose transporters and glycolytic enzymes (Liu et al., 2014), while promoting MDSC differentiation into TAMs (Corzo et al., 2010).

### Amino Acid Metabolism

Another essential nutrient that supports the energy demand of MDSCs is amino acids. Glutamine is a primary nitrogen source and the second most utilized carbon source for energy production (following glucose). It plays an important role in MDSC maturation and immunosuppressive function. A recent report studied the role of a small molecule glutamine antagonist, 6-diazo-5-oxo-l-norleucine (DON) on MDSCs (Oh et al., 2020). DON limits glutaminolysis, which stimulates apoptosis and reprograms MDSCs toward a pro-inflammatory phenotype with enhanced antigen presentation that stimulates T cell-mediated anti-tumor response (Oh et al., 2020). Supporting this notion, another study found that N-methyl-d-aspartate (NMDA) glutamate transport receptor control the immunosuppressive function of human immature myeloid cells, while glutamine-derived α-ketoglutarate (αKG) is crucial for their expansion (Wu et al., 2019a). Blocking glutamine by bis-2-(5-phenylacetamido-1,2,4-thiadiazol-2-yl) ethyl sulfide (BPTES), a selective inhibitor of glutaminase 1 (GLS1), can abolish the generation of MDSC (Wu et al., 2019a). More interestingly, both studies show that glutaminolysis inhibition can overcome resistance to immune-checkpoint blockade in the mouse 4T1 breast tumor model.

Metabolism of the essential amino acid l-arginine by MDSCs acts as a major immunosuppressive pathway that obstructs T cell activation and function. Elevated expression of ARG1 is a hallmark of MDSCs, which generates urea and l-ornithine from l-arginine (Raber et al., 2012). In addition, inducible nitric oxide (NO) synthase (iNOS)-catalyzed conversion of l-ornithine to NO and l-citrulline is also elevated in MDSCs. Both metabolic pathways contribute to MDSC-arginine deprivation, and can reduce the expression of CD3ζ and limit T cell proliferation (Raber et al., 2012). Furthermore, NO can abolish IL-2 receptor-mediated induction of signal transducer and activator of transcription 5 (STAT5) signaling pathway and promote apoptosis in effector T cells (Bingisser et al., 1998). Two ARG1 inhibitors, NOHA and nor-NOHA, have been tested in various cancer models and can diminish the immuno-inhibitory function of MDSCs and improve anti-tumor T cell response (Geiger et al., 2016). Tryptophan metabolism is a similar amino acid pathway and is required for cell division during T cell activation. However, most intratumoral tryptophan is consumed by IDO1 expressing MDSCs, limiting the availability for T cell metabolism. IDO1 catalyzes tryptophan to produce the immunoregulatory molecule kynurenine. This MDSC-induced intratumoral tryptophan starvation leads to impaired immunity against cancer and has been shown to drive immune escape in several cancer models (Källberg et al., 2010; Curtis et al., 2012; Smith et al., 2012; Yu et al., 2013; Schafer et al., 2016). MDSCs utilize similar mechanisms to restrict cysteine, an amino acid oxidized dimer that regulates DNA and protein synthesis and proliferation. Cysteine is required for cytokine production during T cell activation (Angelini et al., 2002; Castellano and Molinier-Frenkel, 2020) and can only be provided by antigen-presenting cells (APCs) such as macrophages and DCs through the process of importing cystine and converting it into cysteine (Castellano and Molinier-Frenkel, 2020). MDSCs express enhanced levels of x_c_^−^ transporter, which increases cystine uptake (Srivastava et al., 2010) and induces an environment with limited cystine availability. In tumors, this MDSC-mediated cystine deprivation impairs cysteine production by APC and blunts T cell activation (Srivastava et al., 2010).

### Lipid Metabolism

Enriched fatty acids in the TME can also serve as an important alternative energy source for MDSCs and regulate their function. Upon transport into the mitochondria by the membrane protein carnitine palmitoyltransferase 1 (CPT1), fatty acids are oxidized to produce acetyl CoA which enters the oxidative phosphorylation (OXPHOS) and TCA cycles for energy generation. Several studies discovered that tumor-infiltrating MDSCs frequently utilize the fatty acids oxidation process for energy production (Husain et al., 2013; Hossain et al., 2015; Yan et al., 2019). Compared to their splenic counterparts, tumor MDSCs reprogram their lipid metabolic profile by increasing fatty acids uptake and oxidation, which augments mitochondrial mass; increases oxygen consumption rate (OCR); and upregulates key enzymes involved in fatty acid oxidation (FAO), including CPT1, acyl CoA dehydrogenase (ACADM), peroxisome proliferator-activated receptor-gamma coactivator 1-β (PGC1β), and 3-hydroxyacyl-CoA dehydrogenase (HADHA) (Hossain et al., 2015). Drivers of this metabolic profile shift include tumor-derived cytokines (e.g., G-CSF and GM-CSF) that signal through STAT3 and STAT5 pathways to upregulate CD36, a fatty acid transporter (Al-Khami et al., 2017). Furthermore, proto-oncogene serine/threonine kinase (PIM1), a downstream target of STAT3, can activate peroxisome proliferator-activated receptor-γ (PPAR-γ), a master regulator of lipid metabolism and CD36 expression (Xin et al., 2021). Selective inhibition of PIM1 leads to defective fatty acid metabolism, which abolishes the immunosuppressive phenotype of tumor-infiltrating myeloid cells and promotes the effector function of anti-tumor CD8 T cells (Xin et al., 2021). Consequently, this inhibition improves the efficacy of programmed cell death protein ligand 1 (PD-L1) blockade and sensitizes non-responders to immune checkpoint blockade (ICB). Furthermore, lysosomal acid lipase (LAL), a critical lipid metabolizer, stimulates the PPAR-γ pathway and bolsters the ability of MDSCs to stimulate tumor proliferation and migration (Zhao et al., 2016). Inhibition of MDSC FAO reduces production of immunosuppressive molecules and impedes suppression of T cells, which produces a synergistic anti-tumor effect when combined with chemotherapy and adoptive cellular therapy (Hossain et al., 2015; Al-Khami et al., 2017).

Several other lipid metabolic pathways also impact the intra-tumoral immunosuppressive phenotype of MDSCs. A recent study identified that the low-density lipoprotein (LDL) receptor lectin-type oxidized LDL receptor 1 (LOX-1) is a specific marker for human intratumoral PMN-MDSC (Condamine et al., 2016; Chai et al., 2019). LOX-1^+^ cells exhibit the enriched gene signature and immunomodulatory activity of suppressive PMN-MDSCs (Condamine et al., 2016; Chai et al., 2019). As a key regulator of lipid homeostasis, liver X receptor (LXR) can drive the expression of apolipoprotein E (ApoE), which binds to LDL receptor to induce the transportation of lipoprotein particles and lipoprotein metabolism (Laffitte et al., 2001; Hong and Tontonoz, 2014). Activation of this pathway by LXR agonists increases apoptosis-mediated MDSC depletion, which delayed tumor progression in B16-F10 melanoma tumor-bearing mice (Tavazoie et al., 2018). On the contrary, genetic deletion of ApoE obstructs the accumulation of MDSCs and blocks their suppressive activity against T cell proliferation (Tavazoie et al., 2018). This LXR/ApoE axis in MDSCs has also been associated with a suppressed anti-tumor T cell responses in ovarian cancer and melanoma (Tavazoie et al., 2018). Another example is the metabolism of the polyunsaturated fatty acid arachidonic acid, which can be converted by COX-2 into prostaglandins such as prostaglandin E2 (PGE2). PGE2 can promote MDSC accumulation and suppress NK cell cytotoxicity *via* activating p38MAPK and ERK-mediated production of TGFβ (Mao et al., 2014). It also has been shown that tumor-derived PGE2 increases nuclear accumulation of p50 NF-κB, which augments NO-mediated immunosuppression (Porta et al., 2020). Altogether, these data suggest that lipid metabolism plays an important role in MDSC phenotype and targeting this pathway may limit the immune-inhibitory functions of MDSCs.

## TAM

TAMs are one of the most abundant TIMs, and have been extensively described in various solid tumors. Recent studies revealed that TAMs can replenish from either circulating monocytes of hematopoietic origin or tissue-resident macrophages of embryonic origin. In a simplified view, TAMs are comprised of two phenotypically and functionally distinct subsets. Anti-tumorigenic TAMs are similar to M1-like macrophages stimulated by toll-like receptor (TLR) ligands and interferon-γ (IFN-γ), which produce ROS and NO and secrete pro-inflammatory cytokines (e.g., IL-1β, IL-6, TNF-α), as well as IL-12 and IL-23, and chemokines such as C-X-C motif ligand 9 (CXCL9) and CXCL10. Their primary functions are activating anti-tumor immune response and direct phagocytosis of cancer cells. In contrast, pro-tumorigenic TAMs closely resemble IL-4- and IL-13-induced anti-inflammatory M2-like macrophages. Pro-tumorigenic TAMs produce an array of inhibitory molecules such as ARG1, IL-10, and IDO that exert immunosuppression functions, and angiogenesis factors, such as VEGF and TGF-β that promote tumor progression. These TAM subsets co-exist in the same tumor and play various roles at different stages of oncogenesis. M1 macrophage-mediated chronic inflammation promotes the growth of epithelial cells that can spontaneously acquire cancer-associated mutations and enhance transformation during the cancer-initiating stage. In contrast, the late-stage tumor microenvironment fosters anti-inflammatory TAMs. The majority of TAMs from late-stage tumors behave in an M2-like manner to suppress anti-tumor immunity and have been shown to contribute to therapeutic resistance (DeNardo and Ruffell, 2019; Pinto et al., 2019; Pan et al., 2020; Cendrowicz et al., 2021).

Macrophage subsets acquire distinct metabolic programs that support their different energy demands and regulate the expression of pro- or anti-inflammatory genes. For instance, TLR and IFN-γ induce M1 macrophages to engage in aerobic glycolysis and exit the TCA cycle, which leads to the accumulation of an intermediate metabolite, succinate. Succinate can stabilize HIF-1α, which activates glycolytic gene transcription and reinforces the glycolytic metabolism in M1 macrophages. In M2-like macrophage, IL-4 induces expression of PPARγ, which transcriptionally upregulates genes associated with FAO and mitochondrial biogenesis. Thus, M2 macrophages exhibit more metabolic flexibility since they can rely on OXPHOS with intact TCA using glutamine and fatty acids. However, these oversimplified models cannot fully recapture the highly dynamic and heterogeneous TAMs, which exhibit a complex spectrum of metabolic and functional profiles across different types of tumors or different malignant lesions from the same patient.

### Glucose Metabolism

**FIGURE 3:**
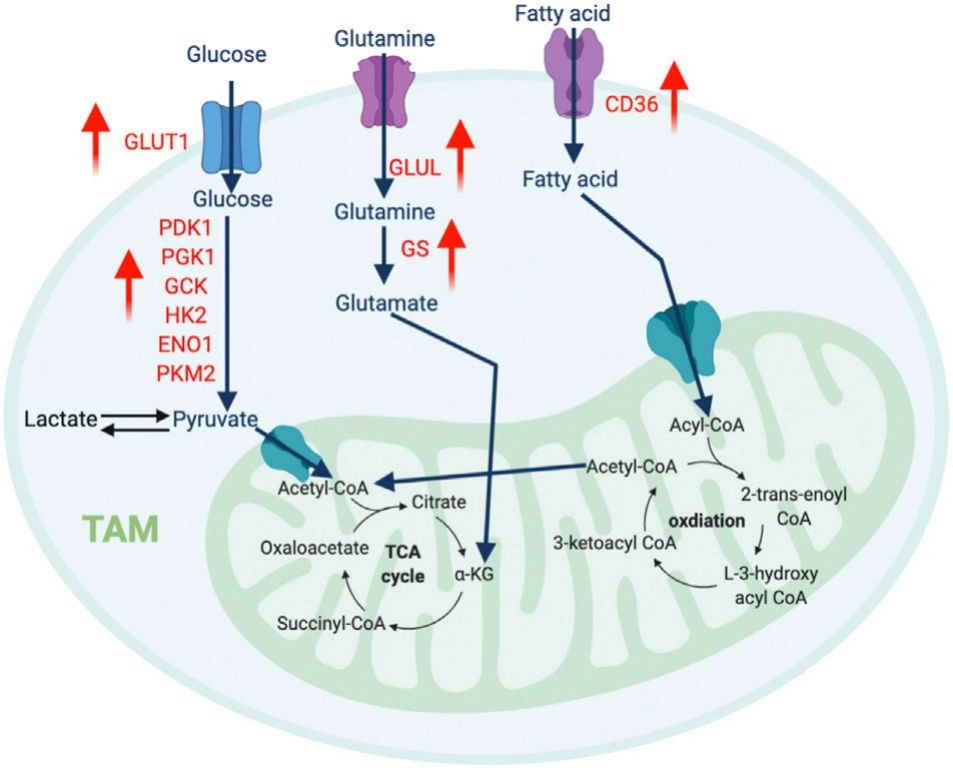
The metabolic features of TAM. The tumor-infiltrating TAMs upregulate glycolysis pathway to regulate their migration and pro-metastatic capability, while promote amino acid and fatty acid metabolism to support their immunosuppressive function. “↑” indicates “increase.” PDK1, Pyruvate Dehydrogenase Kinase 1; PGK1, Phosphoglycerate Kinase 1; GCK, Glucokinase; HK2, Hexokinase 2; ENO1, Enolase 1; PKM2, Pyruvate kinase isozymes M1/M2; GLUT1, Glucose transporter 1; GLUL, Glutamate-Ammonia Ligase; GS, Glutamine synthetase.

Unlike the traditional M2-like macrophages, TAMs depend on glycolysis for metabolism. TAMs derived from circulating monocytes extravasate from the oxygen-rich bloodstream into the oxygen-depleted TME in response to chemokines and pro-inflammatory signals. During this transition, the gradual decrease in oxygen availability induces HIF-1α expression, which transcriptionally induces glycolysis pathway genes such as pyruvate dehydrogenase kinase 1 (PDK1), phosphoglycerate kinase 1 (PGK1), glucose transporter 1 (GLUT1), glucokinase (GCK), and PKM2. Consistently, the per-cell glucose uptake ability of TAMs is significantly higher than tumor cells and other immune cells (Griguer et al., 2005). Comprehensive proteomics analysis discovered several key glycolytic enzymes, such as HK2, PFK, PKM2, and enolase1 (ENO1), are elevated in intratumoral macrophages in the MMTV-PyMT breast cancer and Lewis lung carcinoma (Semba et al., 2016; Liu et al., 2017a). Overall, this evidence suggests that accelerated glycolysis is a characteristic feature of TAMs (Figure 3).

Interestingly, the increased glycolysis in TAMs has been shown to modulate their migration and pro-metastatic capability. HIF1-driven glycolysis in TAMs enables them to migrate more rigorously to the hypoxic regions of the TME. Supporting this notion, inhibiting glycolysis with dichloroacetic acid abolished macrophage migration and accumulation (Semba et al., 2016). Additionally, glycolytic TAMs secrete several growth factors and chemokines, such as VEGF, adrenomedullin (AMD), CXCL8, and CXCL12 activate and recruit endothelial cells, which promotes angiogenesis and metastasis (Chen et al., 2011; Henze and Mazzone, 2016). Using *in vitro* tumor-conditioned macrophages, two studies demonstrated that TAMs with enriched glycolytic signatures support vascular network formation and extravasation of tumor cells (Chen et al., 2011; Arts et al., 2016). This metastatic-promoting ability can be blocked by suppressing glycolysis activity *via* HK2 inhibition.

### Amino Acid Metabolism

An increasing number of reports highlight the fundamental influence of amino acid metabolism on macrophage polarization. Glutamine catabolism fuels M2 macrophages *via* the OXPHOS and TCA cycles. Besides this bioenergetic regulation, elegant immunometabolism research identified that the glutaminolysis-derived metabolite, α-ketoglutarate (αKG) induces an epigenetic landscape favoring the differentiation of anti-inflammatory macrophages. αKG can serve as a vital co-factor for Jumonji domain-containing 3 (Jmjd3)-mediated demethylation of histone H3 at lysine 27 (H3K27). Increased trimethylation of histone H3 at lysine 27 (H3K27me3) is a repressive epigenetic mark dramatically reduces the expression of M2 marker genes. Meanwhile, αKG also promotes the prolyl hydroxylase (PHD)-mediated post-translational modification of inhibitor of nuclear factor kappa-B kinase subunit beta (IKKβ), which disrupts the NF-κB pathway to limit M1 polarization (Liu et al., 2017b). Interestingly, TAMs from cancers such as glioblastoma overexpress glutamine synthase (GS), which is the only human enzyme known to produce glutamine from glutamate. Lack of GS in TAMs strongly favors histocompatibility complex class II (MHC-II)^high^ and CD206^low^ M1-like phenotype with an improved quantity of anti-tumor CD8 T cells (Palmieri et al., 2017). Another key enzyme related to the transport and synthesis of glutamine is glutamate ammonia ligase (GLUL), which supports M2 differentiation (Palmieri et al., 2017). Blocking GLUL can switch the M2-like TAMs into M1 phenotype by reprogramming the glycolysis profile (Palmieri et al., 2017). Overall, glutamine production fosters a pro-tumor M2-like phenotype in TAMs.

Similar to MDSCs, modulation of arginine and tryptophan metabolism in TAMs strengthens their immunoregulatory role supporting tumor immune escape. M2-like macrophages highly express ARG1, which limits arginine in the TME, impairs T-cell receptor (TCR) signaling, and reduces T cell metabolic flexibility. TAM-mediated arginine depletion forces T cells to switch their primary energy source to glucose, the majority of which is consumed by cancer cells and TAMs. Genetic ablation of *Arg1* in macrophages or blocking ARG1 *in vivo* can restrain tumor growth and boost the anti-tumor efficacy of adoptive cell transfer (ACT) therapy in EG7 lymphoma and 3LL lung carcinoma in mice (Rodriguez et al., 2004; Marigo et al., 2016). Unfortunately, this effect has not been observed in humans, as ARG1 is preferably expressed in human granulocytes and ARG1 blockade fails to abolish the immunosuppressive activity of TAMs. Interestingly, iNOS metabolism paradoxically exhibits both anti- or pro-tumoral properties under various conditions, including ROS levels, NO concentration, cancer type, and TME immune landscape (Vannini et al., 2015; Fionda et al., 2016). Enhanced iNOS-induced NO production in LPS-stimulated M1 macrophages can react with ROS to produce peroxynitrite, a strong oxidative and nitrosative agent that induces DNA damage and is associated with macrophage-mediated cytotoxicity (Cifone et al., 1995; Dong et al., 1995; Weiming et al., 2002). In early stages of tumorigenesis, macrophages produce high levels of NO that activate apoptosis in tumor cells (Rahat and Hemmerlein, 2013). Supporting these observations, iNOS expression in macrophages positively correlates with anti-tumor activity in ovarian and pancreatic cancers (Xu et al., 1998; Müerköster et al., 2004). However, a contrasting tumor-promoting role of iNOS in TAMs has also been suggested (Vannini et al., 2015). In well-established or late stage of tumor, the TME is skewed towards anti-inflammatory cytokines, such as TGFβ, which can downregulate the expression of iNOS (Rahat and Hemmerlein, 2013). The arginase pathway is also enriched in TAMs from late-stage tumors, and thus competes with the iNOS pathway for the common substrate l-arginine; as such, the production of NO is significantly reduced (Gordon and Martinez, 2010). This low level of NO can induce angiogenesis which supports tumor growth and metastasis *via* enhancing pro-angiogenic factors, including VEGF and matrix metalloproteinases (Chowdhury et al., 2012; Vannini et al., 2015). In addition, NO exerts immunosuppressive pressure by abolishing the production of IL-12 by macrophages and dendritic cells (Xiong et al., 2004; Wink et al., 2011). Finally, IDO is upregulated in TAMs from various human tumors (such as lung cancer) and can blunt proliferation and cytokine production in activated T cells (Zhao et al., 2012; Meireson et al., 2020). Despite the success in preclinical models and early phase trials, a recent randomized phase III study of IDO inhibitors failed to produce encouraging clinical results in metastatic melanoma patients (Van den Eynde et al., 2020).

### Lipid Metabolism

Macrophages are specialized phagocytic cells with the ability to ingest various forms of lipids, including long-chain fatty acids (LCFAs) and oxidized lipoproteins, and many studies have confirmed that TAMs exhibit increased triglyceride uptake through CD36 and enhanced FAO. Since the TME is enriched with fatty acids, fatty acid metabolism likely plays a pivotal role in TAM polarization. The anti-tumor activity of TAMs is associated with the epidermal fatty acids-binding protein (E-FABP), a lipid chaperone that induces lipid droplet formation and IFN-β production. As a result, E-FABP-expressing macrophages promote recruitment of anti-tumor immune cells, including CD8 T cells and NK cells to inhibit tumor initiation (Zhang et al., 2014). However, a recent study identified diacylglycerol O-acyltransferase (DGAT) as a vital enzyme in macrophage transport of fatty acids into lipid droplets. DGAT inhibitors that block lipid droplets formation and restrict the suppressive activity and level of M2 marker CD206 in TAMs. This phenotype fosters an improved CD8 T cell responsiveness, and delays growth of established tumors. Similarly, other studies discovered that CD36-driven fatty acid accumulation and oxidation enhanced phosphorylation of STAT6, which is the master transcriptional regulator of M2 signature genes (Su et al., 2020). CD36 deficiency in macrophages repolarizes them to an M1-like anti-tumor TAM that can suppress growth and progression of tumors *in vivo* (Su et al., 2020). Overall, lipid metabolism can play opposing roles in determining the pro- or anti-tumor phenotype of TAMs at different tumor stages.

Furthermore, lipid content has a differential impact on the function of TAMs. Macrophages enriched with polyunsaturated fatty acids (PUFA) and linoleic acid (18:3) exhibit cytotoxic activity against P815 tumor cells, while this effect is absent in macrophages enriched with saturated stearic acid (18:0) (Schlager et al., 1983). In PDAC, tumor-derived arachidonic acid induces TAMs to produce immunoinhibitory and tumor-supporting molecules such as PGE2, VEGF, monocyte chemoattractant protein-1 (MCP-1), IL-6, and matrix metallopeptidase (MMP)-9. The direct pro-tumorigenic functions of PGE2 stimulates angiogenesis and enhances the proliferation and migration of cancer cells. Additionally, PGE2 triggers a signal transduction cascade that upregulates PD-L1 expression in TAMs, which abolishes the effector function of cytotoxic T cells. Aside from favoring M2-like polarization, arachidonic acid also impacts recruitment of monocyte-derived macrophages. 5-lipoxygenase, an enzyme that metabolizes arachidonic acid, can promote the secretion of chemokine CCL2, which engages CCR2 on monocytes and macrophages to induce infiltration into human renal cell carcinoma (Daurkin et al., 2011).

## TADC

In the TME, DCs are the primary antigen-presenting cell and specializes in priming different types of effector T cells; they are one of the most important predictors of response to immunotherapy. TADCs capture and process tumor antigens, upregulate the expression of MHC-peptide complexes and costimulatory molecules, and secrete cytokines. DC-mediated antigen presentation is essential for a productive immune response against cancer cells. Upon activation, DCs enter glycolytic flux within minutes to support the high biosynthetic requirements associated with antigen presentation. Blocking this early activation of glycolysis can dramatically dampen multiple functions of DCs, such as antigen presentation and expression of cytokine stimulatory molecules. Since glucose deprivation is predominant in tumors, these tumor-infiltrating DCs may exhibit bioenergetic defects. However, the detailed mechanism and impact of nutrient limitation in TADC metabolism and function remain largely unexplored. Due to the highly glycolytic nature of tumor cells, intratumoral lactate accumulation can activate G-protein-coupled receptor (GPR81) in DC (Brown et al., 2020). As a result, the production of cyclic adenosine monophosphate (cAMP), IL-6, and IL-12 are inhibited, which dampens immune response and accelerates tumor growth (Raychaudhuri et al., 2019).

**FIGURE 4:**
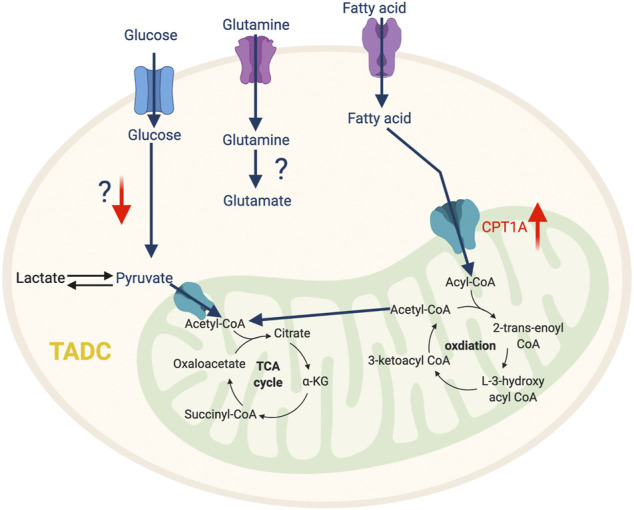
The metabolic features of TADC. The tumor-infiltrating TADC upregulate fatty acid metabolism to support their immunosuppressive function while the role amino acid and glycolysis pathway in not yet clear. “↑” indicates “increase.” CPT1A, Carnitine Palmitoyltransferase 1A.

Besides glycolysis, fatty acid metabolism also significantly impacts DC phenotype. In ovarian cancer and hepatocellular carcinoma, the key enzyme of *de novo* lipogenesis, fatty acids synthase (FASN), is overexpressed and produces an excessive amount of fatty acids, which can be ingested by DC *via* scavenging receptors (macrophage scavenger receptor 1 and CD204) and stored inside DC as lipid droplets (Jiang et al., 2018; Hu et al., 2020b). This fatty acid accumulation impairs transportation of peptide-MHC class I complexes to the cell surface and reduces the level of costimulatory molecules and cytokines, which weakens the ability of TADCs to stimulate an anti-tumor T cell response (Jiang et al., 2018). Furthermore, the accumulation of fatty acids promotes FAO, which is the preferred metabolic pathway of tolerogenic DCs, an important driver of tumor-mediated immune evasion. The tumor-derived Wnt5 ligand can activate β-catenin signaling in TADC, which upregulates PPARγ to induce Carnitine Palmitoyltransferase 1A (CPT1A)-driven FAO in a murine melanoma model (Zhao et al., 2018a). Enhanced FAO in TADC favors the differentiation of T regulatory cells (Tregs) and inhibits anti-tumor T cell activation (Figure 4). Using etomoxir to inhibit FAO decreases the ability of Wnt5-exposed DCs to expand Tregs, which enhances anti-tumor immunity and anti-PD-1 antibody immunotherapy efficacy (Zhao et al., 2018a). Consistently, mice with specific deletion for β-catenin in CD11c^+^ cells exhibit reduced numbers of IL-10-producing Tregs intratumorally, which is associated with enhanced anti-tumor CD4 and CD8 T cell response (Zhao et al., 2018a).

## TAN

**FIGURE 5:**
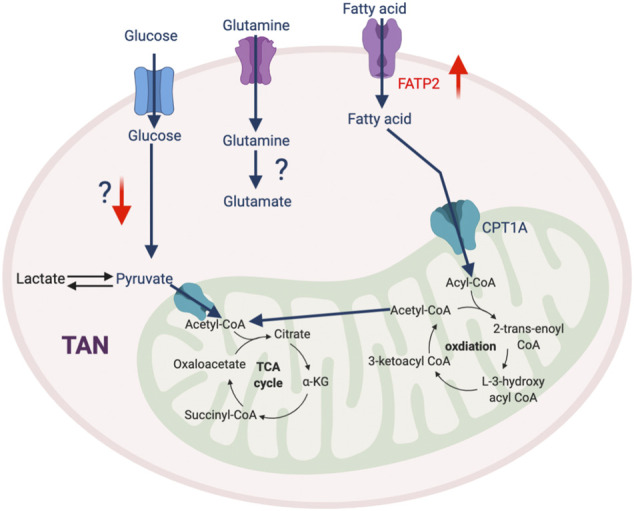
The metabolic feature of TAN. Most metabolic pathways in TAN are not well-defined, except that FATP2-mediated fatty acid metabolism plays an important role in their pro-tumorigenic phenotype. “↑” indicates “increase.” FATP2, Fatty acid transport protein 2.

In addition to the traditional roles that neutrophils play in mediating the immune response to infection, accumulating evidence reveals that TAN-mediated immunosuppression also contributes to therapeutic resistance of tumors. At the early stages of tumorigenesis, neutrophils are recruited to the TME with elevated oxidative phosphorylation and glycolysis to meet their energy demand (Patel et al., 2018). This metabolic flexibility is impaired by the TME, which induces TANs to rely on oxidative phosphorylation *via* the c-Kit singling pathway (Rice et al., 2018). This metabolic reprogramming augments mitochondrial function, which enhances ROS production and stimulates inhibitory activity against T cells (Rice et al., 2018). A recent study described that high mitochondrial content and potential in neutrophils is required to impede T cell function (Volberding et al., 2021). PIM1 mitochondrial kinase, which promotes mitochondrial fusion and fitness, is highly expressed in TANs, and blocking PIM1 releases T cells from neutrophil-mediated immunosuppression (Volberding et al., 2021). Another report identified that GM-CSF signaling *via* STAT5 promotes the expression of long-chain fatty acids transport protein 2 (FATP2) (Veglia et al., 2019) and enhances the accumulation of arachidonic acid leading to excessive production of PGE2, which potentiates suppressive properties of TANs. Thus, selective pharmacological inhibition of FATP2 could abolish neutrophil-mediated immunosuppression and retard tumor growth either alone with combined with ICB (Veglia et al., 2019). Overall, the detailed mechanism of metabolic adaptation in TANs and its impact on cancer are still unclear and warrant further investigation (Figure 5).

## Clinical Implication of Targeting TIM’s Metabolism

Metabolic regulation is increasingly recognized as the major factor determining pro or anti-tumorigenic function of TIMs. Therefore, the metabolic reprogramming of TIMs may unleash anti-cancer immunity and augment available therapeutic modalities. Such approaches are supported by many immunometabolism studies mentioned and have gained substantial interest. Although there are limited strategies to specifically target TIM metabolism, numerous clinical trials have assessed the feasibility of generic metabolic modulators to combat cancer (Table 1).

**TABLE 1:** Metabolic modulators in clinical trials.

Pathway	Drug	Cancer type	Effect on metabolism	Clinical trials
Glycolysis	2-Deoxy-d-glucose	advanced solid tumors	Inhibiton of glycolysis pathway	NCT00096707 Raez et al. (2013)
	shikonin	Bladder Cancer	Inhibiton of PKM2	Yeh et al. (2015); Wang et al. (2018b); Durchschein et al. (2018)
		Lung cancer		
		Melanoma		
	PX-478	advanced solid tumors or lymphoma	Inhibiton of HIF-1a	NCT00522652 Palayoor et al. (2008)
				NCT01120288
	CB-1158	Advanced-stage solid I/II tumors	Inhibits arginase	NCT02903914 NCT03910530 NCT03314935 NCT03837509 NCT03361228
	ADI-PEG 20 (PEGylated arginine deiminase)	Advanced-stage solid I tumors	Inhibits arginase	NCT03254732
Amino acid	CB-839	Colorectal cancer, NSCLC, renal cell carcinoma, melanoma	Inhibition of glutamin metabolism (increased dependence of glutamine in cancer cells)	NCT03263429, NCT03831932, NCT02771626
	Linrodostat (BMS-986205; IDO1 inhibitor)	Advanced-stage cancers I/II	Inhibits IDO	NCT03459222
	Enasidenib AG-221, AG-120 (Ivosidenib), AGI-5198, AG-881	Acute myeloid leukaemia, bile duct cancer, glioma, haematological malignancies, solid tumours	Inhibition of α-KG reduction to 2-HG by mutant IDH leading to impaired demethylation	NCT02074839 Urban et al. (2017); Golub et al. (2019)
				Enasidenib and ivosidenib approved for acute myeloid leukaemia
Lipid metabolism	Metformin	Type II diabetes, cancer in general, rheumatoid arthritis	Reduction in glycolytic pathway, reduced glucose blood levels, increased FAO, inhibition of respiration, inhibition of mTOR	NCT02019979, NCT02640534, NCT01310231, NCT02312661
	Paclitaxel, Methotrexate, Doxorubicin	Multidrug resistant cancer	Impaired efflux leading to accumulation	Studies ongoing e.g., phase III trial for breast cancer. Costantini et al. (2010), NCT02488967
	Aspirin (COX1 and/or COX2 inhibitor) or celecoxib (COX2 inhibitor)	Advanced-stage MSI-H/dMMR cancers	Inhibitors of COX enzymes and/or PGE2 signaling	NCT03638297

Glycolysis plays an important role in the migration and accumulation of immunosuppressive TIMs, and altering intratumoral glycolysis may improve anti-tumor response. For example, the glucose analogue 2-deoxy-d-glucose (2-DG) can competitively suppress glycolysis, which can abolish TIM-mediated immunosuppression. This approach is being tested in phase I clinical trials alone or in combination with docetaxel against advanced solid tumors (Raez et al., 2013). PKM2, mediates the last step of glycolysis, which supports the pro-tumorigenic phenotype in MDSC and TAM; its expression is being examined as a biomarker of metabolic reprogramming in patients with intracranial tumors or recurrent glioblastoma (NCT03539731). Interestingly, treatment with PKM2 blocker shikonin can overcome chemotherapeutic resistance in lung, melanoma, and bladder cancer (Wang et al., 2018a; Zhao et al., 2018b). Another important regulator of glycolysis in TIM is HIF1α, which transcriptionally activates the genes involved in the glycolysis pathway. Pharmacological HIF1α inhibitor PX-478 demonstrates potent anti-tumor efficacy in various pre-clinical cancer models and is being evaluated in a phase I trial in patients with advanced solid tumors or lymphoma (NCT00522652). Furthermore, treatment with an antisense oligonucleotide inhibitor of HIF1α has shown clinical benefit in patients with advanced solid tumors (NCT01120288) (Wu et al., 2019b). However, off-target side effects of these approaches can become a challenge because of the essential role of glycolysis in normal cells.

As previously discussed, myeloid cells from tumors also employ amino acid metabolic pathways to exert their function. Reagents blocking ARG1 activity, such as CB-1158 and ADI-PEG 20, can dampen the TIM-induced immune evasion and inhibit tumor progression. Several clinical trials are testing CB-1158 as a single agent or in combination with ICB (NCT02903914, NCT03910530, NCT03314935, NCT03837509, NCT03361228), and an additional phase I trial is assessing the synergic effect between ADI-PEG 20 and pembrolizumab (NCT03254732). Targeting amino acid metabolism with an IDO inhibitor showed no clinical promise in a phase III trial of metastatic melanoma patients (Raber et al., 2012). Finally, a potent, selective inhibitor of glutaminase, CB-839 is being tested in patients with colorectal cancer, non-small cell lung cancer (NSCLC), renal cell carcinoma, and melanoma (NCT03263429, NCT03831932, NCT02771626).

Targeting lipid metabolism may also switch TIMs toward an anti-tumorigenic phenotype. Metformin is used to treat hypoglycemia, but can also promote FAO to reduce the proportion of M2-like TAMs and increase M1-like TAMs. Several clinical trials are examining the benefit of metformin alone or in combination with carboplatin/paclitaxel to treat prostate, breast, ovarian and NSCLC (NCT02640534, NCT02019979, NCT01310231, and NCT02312661). Blocking the TCA cycle by reducing the intermediate metabolite α-KG is another approach to interrupt fatty acid metabolism. α-KG inhibitors such as enasidenib (AG-221), ivosidenib (AG-120), AGI-5198, and AG-881 are being evaluated in various hematological malignancies and solid tumors (NCT02074839). More recently, aspirin and celecoxib, which block the polyunsaturated fatty acid pathway mediator COX-2, are being tested in advanced-stage colorectal cancers (NCT03638297). However, designing studies that target fatty acid metabolism must consider the caveat that anti-tumor T cells utilize similar metabolic pathways for survival and function. Several elegant studies have reported that mitochondrial function is vital for exhausted CD8 T cells to sustain their effector activity such as producing IFNγ and granzyme B (Yu et al., 2020; Dumauthioz et al., 2021). Therefore, these FAO inhibitors may have a detrimental effect on CD8 T cells, reducing their anti-tumor efficacy.

## Future Perspectives and Concluding Remarks

Recent immunometabolism research provides appealing evidence to suggest that metabolic regulation plays an important role in the differentiation and function of TIMs. This bioenergetic profile can be influenced by tumor cells in various ways. First, tumor cells alter nutrient availability, such as glucose deprivation and lipid enrichment, which induces metabolic reprogramming in myeloid cells. Secondly, tumor-derived factor, metabolic byproduct and waste such as hypoxia and lactate, can modulate signaling pathways to induce a metabolic shift in TIMs. This metabolic rewiring can subvert the anti-tumor function of TIM and promote the pro-tumorigenic phenotype. These discoveries provide a unique opportunity to selectively modulate myeloid cell metabolism to fight cancer.

**FIGURE 6:**
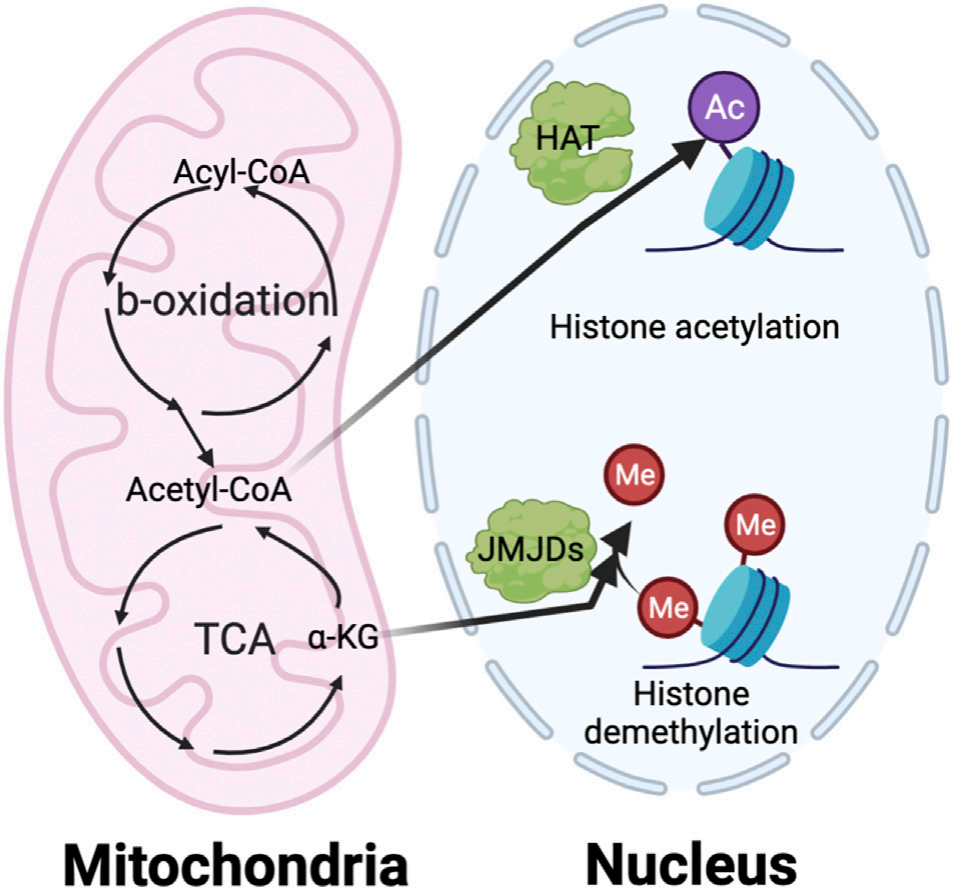
The interaction of metabolism and epigenetics. The intermediate metabolites in metabolic pathway play a vital role in regulating the epigenetic modification, especially histone acetylation and methylation. The histone acetylation requires acetyl-CoA generated from TCA cycle in mitochondria as an essential substrate. On the other hand, the histone demethylation needs a-KG from TCA cycle as important cofactor of the Jumonji C domain-containing histone demethylases (JMJDs).

Although metabolic targeting holds great promise as an anti-cancer therapeutic approach, several challenges need to be addressed. One is an improved understanding of the underlying mechanisms of metabolic regulation. Many elegant studies clearly show that the metabolic switch between the different phenotypes of myeloid cells both satisfies the biogenetic need and impacts epigenetic regulation of gene expression (Dai et al., 2020). It is well-appreciated that many intermediate metabolites, such as acetyl-coA and α-KG, serve as critical donors, substrates, and cofactors for epigenetic modifications (Figure 6). Nevertheless, how nutrients inside tumors are processed through pathways such as glycolysis, FAO, and glutaminolysis, and how these metabolites regulate epigenetic events to specify pro- or anti-tumorigenic function is still under investigation. Another challenge is identifying TIM-specific metabolic regulators. Most current studies use common reagents that broadly target metabolic circuitries shared between TIM and other cells, such as 2-DG and etomoxir. This could lead to paradoxical effects influencing tumor progression. For example, blocking FAO by etomoxir can reduce immunosuppressive TAMs and MDSCs (Hossain et al., 2015; Su et al., 2020); however, anti-tumor CD8 T cells may also rely on fatty acid metabolism to sustain their function (Zhang et al., 2017; Lin et al., 2020). Furthermore, the detail of metabolic interplay between TIMs, other immune components, and malignant cells remains unclear. This prevents us from accurately predicting the result of metabolic intervention since it will depend on net effects from multiple cell compartments. For example, induction of glycolysis in M2-like TAMs has the potential to repolarize them into an M1-like phenotype for promoting immunity against cancer. However, it is easy to foresee that such glycolysis activation in TAM can deprive immune effector cells of glucose, which may lead to immunosuppression. The heterogeneity of bioenergetic metabolism across various TIM populations makes it hard to design effective metabolic targeting approaches. Future studies elucidating the impact of metabolism on epigenetics and TIM-specific metabolic profiles will help to develop novel therapeutic strategies for metabolic reprogramming of TIM.
